# Dermatoskopie von Genodermatosen

**DOI:** 10.1007/s00105-023-05124-7

**Published:** 2023-03-07

**Authors:** Dóra Plázár, Marie Isolde Joura, Norbert Kiss, Márta Medvecz

**Affiliations:** Klinik für Dermatologie, Venereologie und Dermatoonkologie, Semmelweis Universität Budapest, Mária Straße 41, 1085 Budapest, Ungarn

**Keywords:** Ichthyose, Akantholytische Dermatose, Dyskeratose, Pseudoxanthoma elasticum, Angiokeratom, Ichthyosis, Acantholytic dermatosis, Dyskeratosis, Pseudoxanthoma elasticum, Angiokeratoma

## Abstract

Genodermatosen sind eine Gruppe erblicher Hautkrankheiten, deren Diagnose aufgrund ihrer Seltenheit sowie klinischen und genetischen Vielfalt eine Herausforderung darstellt. Die meisten Genodermatosen werden autosomal oder X‑chromosomal vererbt, auch Mosaikformen werden beobachtet. Genodermatosen umfassen verschiedene Phänotypen, die von einer begrenzten kutanen Erkrankung bis hin zu einer schweren kutanen und extrakutanen Beteiligung reichen. Sie können auch Zeichen multisystemischer Störungen sein. Trotz der jüngsten Fortschritte in der Gentechnologie und den bildgebenden Verfahren der Haut stellt die Dermatoskopie für das Screening, die Diagnostik und die therapeutische Nachsorge eine nützliche Untersuchungsmethode dar. Bei der ektopischen Mineralisierungsstörung Pseudoxanthoma elasticum und der lysosomalen Speicherkrankheit Morbus Fabry können die Symptome auf der Haut auf Beteiligungen innerer Organe hinweisen. Bei Keratinisierungskrankheiten wie Ichthyosen und akantholytischen Dyskeratosen wie Morbus Darier und Morbus Hailey-Hailey kann die Dermatoskopie helfen, die Wirksamkeit der Therapie zu zeigen, indem sie Hintergrunderythem, Hyperkeratose und den interkeratinozytären Raum sichtbar macht. Die Dermatoskopie ist eine nichtinvasive In-vivo-Untersuchung, die in der allgemeinen Dermatologie zunehmend an Bedeutung gewinnt und ein nützliches, leicht zugängliches Instrument zur Erkennung charakteristischer Merkmale von Genodermatosen sein kann.

## Abstract (en)

Genodermatoses are a group of inherited skin diseases whose diagnosis is challenging due to their rarity as well as their clinical and genetic diversity. The majority of genodermatoses are autosomal or X‑linked inherited, but mosaic forms are also observed. Genodermatoses comprise various phenotypes ranging from limited cutaneous disease to severe cutaneous and extracutaneous involvement and may also be early warning signs of a multisystemic disorder. Despite recent advances in genetic technology and skin imaging modalities, dermoscopy can be useful for screening, diagnosis, and treatment follow-up. In ectopic mineralization and lysosomal storage disorders (pseudoxanthoma elasticum and Fabry disease, respectively), cutaneous manifestations may indicate involvement of other organs. In keratinization diseases (e.g., ichthyoses) and acantholytic skin fragility disorders (e.g., Darier and Hailey–Hailey disease), dermoscopy may help to assess treatment response by visualizing background erythema, hyperkeratosis, and interkeratinocyte space prominence. Dermoscopy is a noninvasive, easily accessible, useful, in vivo assessment tool that is well established in dermatology to recognize characteristic features of genodermatoses.

Genodermatosen sind eine Gruppe erblicher Hautkrankheiten, deren Diagnose aufgrund ihrer Seltenheit, klinischen und genetischen Vielfalt schwierig ist. Die Dermatoskopie ist eine nichtinvasive In-vivo-Untersuchung, die sich in der allgemeinen Dermatologie immer mehr durchsetzt und für die Erkennung charakteristischer Merkmale von Genodermatosen nützlich sein kann. Bei hereditären Keratinisierungskrankheiten, akantholytischen Hautfragilitätssyndromen, ektopischen Mineralisierungsstörungen und lysosomalen Speicherkrankheiten demonstrieren wir die Anwendung der Dermatoskopie im diagnostischen Prozess.

## Diagnose von Genodermatosen

Bei den Genodermatosen handelt es sich um eine klinisch und genetisch vielfältige Gruppe vererbter Hautkrankheiten. Die meisten kommen selten vor und betreffen weniger als 1 von 2000 Menschen der Allgemeinbevölkerung. Genodermatosen sind chronische Erkrankungen, deren kutane und extrakutane Manifestationen schwerwiegende Auswirkungen auf den allgemeinen Gesundheitszustand und die Lebensqualität der PatientInnen haben können. Diagnose, Behandlung und Nachsorge erfordern ein hohes Maß an Komplexität nach multidisziplinären Grundsätzen. Der mehrstufige Diagnosealgorithmus für vererbte Hauterkrankungen empfiehlt, bei der Diagnose phänotypische Merkmale und klinische Daten, die Art der Vererbung, Zielproteine und genetische Variationen zu berücksichtigen [27].

Die Dermatoskopie kann ein nützliches, nichtinvasives, ergänzendes Diagnoseinstrument für die Bewertung charakteristischer Haut‑, Nagel- oder Haarbefunde von Genodermatosen sein [9]. Die Standardisierung der dermatoskopischen Terminologie ist wichtig, um die Reproduzierbarkeit und Vergleichbarkeit der dermatoskopischen Untersuchungen zu gewährleisten. Neben der Standardisierung der dermatoskopischen Terminologie werden in der Literatur auch konkurrierende deskriptive und metaphorische Begriffe verwendet [9, 17].

## Erkrankungen der epidermalen Strukturen

### Störungen der Keratinisierung: Ichthyosen

Erbliche Ichthyosen, die auch als Mendelsche Verhornungsstörungen bezeichnet werden, sind eine genetisch und klinisch heterogene Gruppe, die sich durch Hyperkeratose, diffuse Schuppung, Xerose und Erythrodermie unterschiedlichen Grades zeigen.

Der Schweregrad der Symptome variiert stark aufgrund von Defekten der epidermalen Barriere und verschiedenen Störungen des terminalen Differenzierungsprozesses der Keratinozyten. Nichtsyndromale Ichthyosen können von syndromalen Ichthyosen unterschieden werden. Die Ichthyosis vulgaris (IV, OMIM # 146700) ist der häufigste Typ und wird durch autosomal-semidominante Mutationen des Filaggrin-Gens (*FLG*) verursacht. Zu den klinischen Merkmalen gehören Hyperlinearität der Handflächen, Keratosis pilaris sowie eine feine oder ausgeprägte Schuppung am unteren Rumpf und an den Extremitäten. IV geht häufig mit Atopie einher [28]. Das dermatoskopische Bild einer IV zeigt ein ausgeprägtes lineares Muster, erhabene oder ausgefranste Keratinozytenränder mit einem Hintergrunderythem [25]. Ein kreuz und quer verlaufendes Muster aus feinen weißen Schuppen wurde ebenfalls beschrieben. Dieses Muster stellt die Hyperkeratose im Rahmen einer zunehmenden Xerose dar [3, 11].

**Figure 1:**
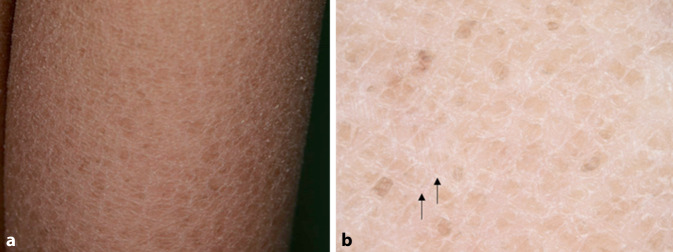

Die X‑chromosomal-rezessive Ichthyose (XRI, OMIM # 308100) tritt fast ausschließlich bei männlichen Patienten auf. Sie ist die Folge eines Steroidsulfatasemangels und wird durch eine Deletion oder Mutation des* STS*-Gens auf Chromosom Xp22.31 verursacht. Klinisch zeichnet sich XRI durch ausgedehnte, dunkelbraune, polygonale Schuppen besonders am Stamm und an den Streckseiten der Gliedmaßen aus, wobei die großen Beugen nicht betroffen sind (Abb. 1a; [28]). Dermatoskopisch zeigt sich bei XRI ein rhomboides oder mosaikartiges Muster aus braunen Strukturen mit Zwischenräumen (Abb. 1b; [11]).

**Figure 2:**
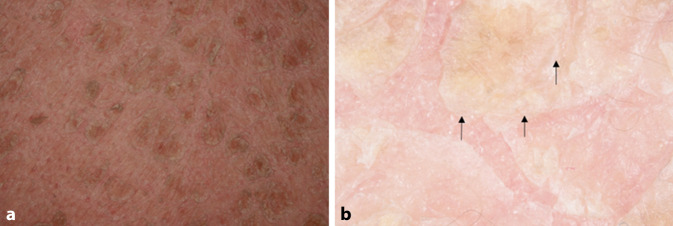

Die autosomal-rezessive kongenitale Ichthyose (ARCI) ist klinisch und genetisch sehr heterogen. Betroffene Neugeborene kommen häufig mit einer Kollodiummembran auf die Welt. Aufgrund des umgekehrten Verhältnisses zwischen dem Schweregrad der Ichthyose und der Erythrodermie sind die wichtigsten Hautphänotypen die lamelläre Ichthyose (LI) und die kongenitale ichthyosiforme Erythrodermie (CIE), obwohl es zu phänotypischen Überschneidungen kommen kann. Die LI ist durch eine generalisierte, große, anhaftende, dunkle Schuppung mit leichtem Erythem gekennzeichnet (Abb. 2a; [28]). Es lassen sich viereckige bräunliche Strukturen mit feinen weißen Schuppen beobachten, die in einem lamellären Muster angeordnet sind (Abb. 2b; [11]).

Die Dermatoskopie verbessert bei ARCI die Visualisierung unregelmäßiger Keratinozytengrenzen, darunterliegender vaskulärer Ektasien und Erytheme. Die Visualisierung des Hintergrunderythems, der Hyperkeratose und des Hervortretens der interkeratinozytären Räume kann auch helfen, das therapeutische Ansprechen zu verfolgen [25].

### Dyskeratotisch – akantholytische Dermatosen

#### Morbus Darier

**Figure 3:**
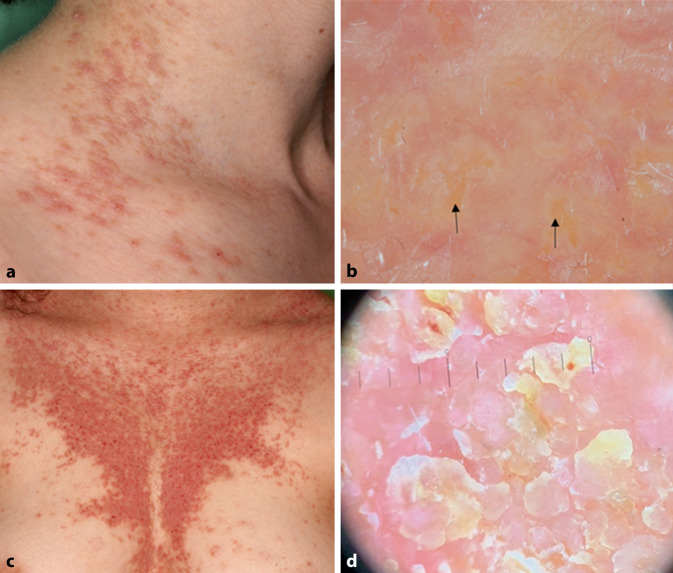

Der Morbus Darier (MD, OMIM # 124200, auch Keratosis follicularis genannt) ist durch einen Verlust der Adhäsion zwischen den Epidermiszellen und eine abnorme Keratinisierung gekennzeichnet. Die autosomal-dominant vererbte Krankheit wird durch heterozygote Mutationen des *ATP2A2*-Gens verursacht, das für eine Kalziumpumpe des endoplasmatischen Retikulums, die sarko/endoplasmatische Retikulum-ATPase Typ 2 (SERCA2), kodiert. Die Krankheit äußert sich in der Regel durch kleine keratotische Papeln v. a. in seborrhoischen Bereichen wie Brust, Rücken, Hals und Gesicht (Abb. 3c). Nagelanomalien, palmare Pits, Schleimhautveränderungen sowie neuropsychiatrische Auffälligkeiten können ebenfalls auftreten. Eine segmentale Form des MD, die durch postzygotische Mutationen (somatischer Mosaizismus) verursacht wird, kann ebenfalls beobachtet werden. Die histopathologische Analyse zeigt Hyper- oder Parakeratose, akantholytische dyskeratotische Zellen, sog. Corps-rounds-Zellen, und suprabasale Spalten [24]. Die Dermatoskopie zeigt zentral gelegene polygonale, sternförmige oder rundlich-ovale gelb-bräunliche Areale, umgeben von einem weißlichen Halo auf einer rosafarbenen, homogenen, strukturlosen Fläche (Abb. 3b). Es können auch weißliche Schuppen und gepunktete oder lineare Gefäße zu sehen sein. In schweren Fällen sind parallele, rötlich-rosa Furchen mit weiß-gelblichen Schuppen oder weißlich-rosa Bereichen verbunden, was ein „reifenprofilartiges“ oder „rissiges flussbettartiges“ Aussehen zur Folge hat (Abb. 3d; [1, 7, 8, 19, 29]).

#### Morbus Hailey-Hailey

**Figure 4:**
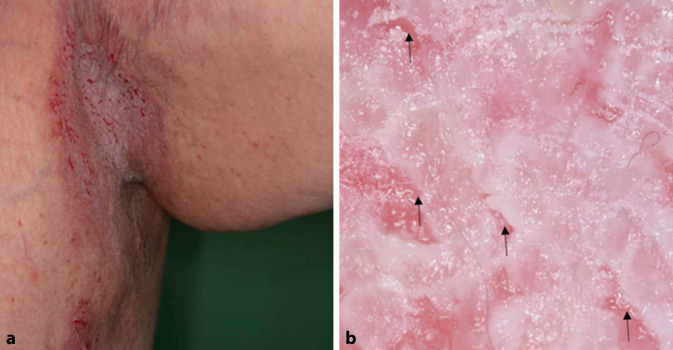

Morbus Hailey-Hailey (MHH, OMIM # 169600, auch bekannt als benigner familiärer chronischer Pemphigus) ist eine autosomal-dominant vererbte akantholytische Genodermatose, die durch heterozygote Mutationen des ATPase secretory pathway Ca^2+^ transporting 1‑Gens (*ATP2C1*-Gen) verursacht wird. Er äußert sich typischerweise durch chronisch rezidivierende, schmerzhafte Erytheme, Bläschen, Erosionen und schuppende erythematöse Plaques, die sich meist in den Beugebereichen der axillären, submammären, inguinalen und perinealen Falten befinden. Oft sind sie symmetrisch verteilt (Abb. 4a). Auch diese segmentale Form wurde beschrieben. Die Histopathologie zeigt eine suprabasale und intraepidermale Spaltbildung. Die Akantholyse ist ausgedehnter als beim MD und betrifft manchmal die gesamte Dicke der Epidermis [24]. Die Dermatoskopie zeigt eine unregelmäßige Kombination weißer und rosafarbener Bereiche in einem „Wolken‑“ oder „Eisbergmuster“ (Abb. 4b; [14]). Weißliche Bereiche sind oft durch rötlich-rosa Furchen getrennt, die wie ein „zerknittertes Gewebe“ aussehen. Es können auch polymorphe Gefäße und Schuppen zu sehen sein [5, 14, 23].

## Mendelsche Bindegewebserkrankungen: Pseudoxanthoma elasticum

**Figure 5:**
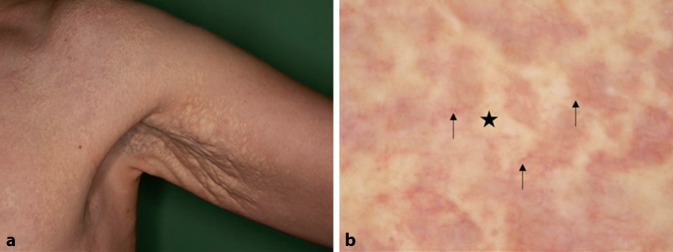

Pseudoxanthoma elasticum (PXE, OMIM # 264800) ist eine multisystemische, autosomal-rezessive Stoffwechselstörung, die durch homozygote oder compound heterozygote Mutationen im *ABCC6*-Gen verursacht wird, das für einen Transmembrantransporter in Hepatozyten kodiert. PXE geht mit einer ektopischen Verkalkung und Fragmentierung der elastischen Fasern in der Haut, den Blutgefäßen und der Netzhaut einher. Die Folge sind eine zunehmende Erschlaffung und ein Elastizitätsverlust der Haut, Arterieninsuffizienz und Netzhautblutungen. Bei der körperlichen Untersuchung zeigen sich multiple und zusammenwachsende asymptomatische, weiche, gelbliche Papeln mit kopfsteinpflasterartigem Aussehen. Sie sind symmetrisch an Hals, Nacken und anderen beugenden Körperstellen verteilt (Abb. 5a). Zu den dermatoskopischen Befunden gehören gelblich-weiße Kügelchen, die zu parallelen oder netzartigen Strängen auf einem leicht purpurroten Hintergrund und oberflächlichen linearen Gefäßen zusammenwachsen können (Abb. 5b; [13, 18, 26]). Der purpurrote Hintergrund kann auf eine subklinische Entzündung hinweisen, die eine erhöhte mikrovaskuläre Perfusion verursacht. Die gelblich-weißen Klümpchen lassen sich durch degenerierte und fragmentierte elastische Fasern und Kalkablagerungen in der mittleren Dermis erklären. Prominente, oberflächliche, lineare Gefäße können die Folge der vaskulären Umstrukturierung sein, die durch die zugrunde liegende Elastolyse der Haut verursacht wird [18].

## Lysosomale Speicherkrankheit

**Figure 6:**
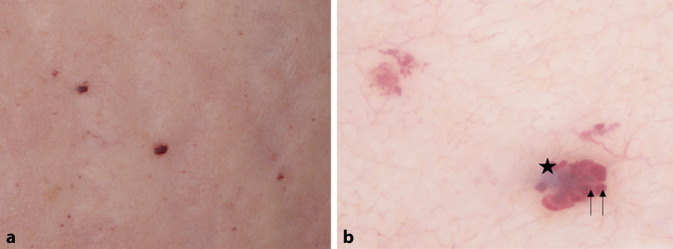

Morbus Fabry (MF, OMIM # 301500, auch bekannt als Angiokeratoma corporis diffusum universale) ist eine X‑chromosomal vererbte lysosomale Speicherkrankheit, die durch eine Vielzahl von Mutationen im *GLA*-Gen verursacht wird. Das führt zu einem Mangel an α‑Galaktosidase A, der eine abnormale und fortschreitende Anhäufung von Glykosphingolipiden insbesondere in Endothelzellen zur Folge hat, was multisystemische Auswirkungen hat. Angiokeratome sind neben Teleangiektasien und Hypohidrose die frühen sowie häufigsten kutanen Symptome des MF. Beim MF treten Angiokeratome als rote oder blauschwarze multiplexe, manchmal gebündelte makuläre oder palpable papulöse Läsionen mit einem Durchmesser von 1–5 mm auf, die oft, aber nicht immer, mit feinen, weißen, keratotischen Schuppen bedeckt sind. Übliche Lokalisationen sind der Rücken und die Vorderseite des Rumpfes, die Gliedmaßen, der Nabel und die Genitalien (Abb. 6a). Die Diagnose ist oft schwierig, insbesondere wenn die Zahl der Angiokeratome gering ist, klinisch relevante oberflächliche Keratosen fehlen oder die Lokalisation ungewöhnlich ist. Die Dermatoskopie zeigt gut abgegrenzte, runde Lakunen, die erweiterte dermale Gefäße und einen weißlichen Schleier darstellen, das Zeichen einer epidermalen Hyperkeratose (Abb. 6b; [4, 12]).

## Diskussion

In den letzten Jahren wurde die Dermatoskopie nicht nur zur Diagnose von melanozytären Läsionen, sondern auch zur Beurteilung weiterer Hauterkrankungen unterschiedlicher Ätiologie einschließlich Genodermatosen eingesetzt. Die Dermatoskopie ist eine nichtinvasive, leicht zugängliche Technik, die eine rasche klinische Beurteilung von Hautläsionen ermöglicht und helfen kann, charakteristische morphologische Merkmale zu erkennen. Dies ist besonders bei Genodermatosen nützlich, da die meisten PatientInnen Kinder sind und es sich als schwierig erweisen kann, in dieser Altersgruppe eine Hautbiopsie durchzuführen. Die dermatoskopische Erkennung von charakteristischen Hautmanifestationen kann die Diagnose bestimmter multisystemischer Erkrankungen erleichtern, darunter die ektopische Mineralisierungsstörung PXE und die lysosomale Speicherkrankheit Morbus Fabry. Bei diesen Erkrankungen kann eine frühzeitige Diagnose zu einer raschen Behandlung der Krankheit und zur Vermeidung von Komplikationen beitragen, da das Vorhandensein von Hautläsionen auf die Beteiligung anderer Organe hinweisen kann [4, 12, 13, 18, 26]. Es ist wichtig zu betonen, dass für die Diagnosestellung neben der dermatoskopischen Beurteilung auch der gesamte klinische Kontext des/der Patienten/Patientin berücksichtigt werden muss, einschließlich des Krankheitsbeginns, der Lokalisation der Hautläsionen, der Familienanamnese und der Ergebnisse der molekularen Tests [27].

Neben der dermatoskopischen Beurteilung muss auch der gesamte klinische Kontext berücksichtigt werden

Auch bei Ichthyosen sowie bei akantholytischen Genodermatosen (MD und MHH) kann die Dermatoskopie genutzt werden, um das Ansprechen auf die Therapie zu verfolgen, indem man das Hintergrunderythem, die Hyperkeratose und das Hervortreten der Interkeratinozytenräume sichtbar macht [5, 7, 8, 14, 19, 23, 25, 29]. Da verschiedene neuartige Therapien für die Behandlung von Genodermatosen erforscht und eingeführt werden, darunter neue und neu ausgerichtete Medikamente, Proteinersatz, Chaperone, Zell- und Gentherapien und andere Ansätze, könnte der Einsatz der Dermatoskopie zur Therapieüberwachung in Zukunft eine ergänzende Anwendung darstellen [22].

Neben der Dermatoskopie wurden weitere neuartige bildgebende Verfahren wie die nichtlineare optische Mikroskopie und die multispektrale Bildgebung als neuartige quantitative Instrumente zur Bewertung von PXE-befallener Haut eingesetzt [10, 15, 21]. Diese Techniken können auch für die Beurteilung einer breiteren Palette von Erbkrankheiten eingesetzt werden, darunter das Ehlers-Danlos-Syndrom, keratinopathische Ichthyose und das Marfan-Syndrom [2, 6, 16, 20]. Die multimodale Bildgebung mit der Kombination von Dermatoskopie und verschiedenen bildgebenden Verfahren könnte ein weiterer Ansatz für die gründliche Untersuchung der Haut von PatientInnen mit Genodermatosen sein, der in der Diagnostik und Therapieüberwachung dieser Erkrankungen eingesetzt werden kann.

## Fazit für die Praxis

- Die Dermatoskopie ist eine nichtinvasive, leicht zugängliche Technik, die eine rasche klinische Beurteilung von Hautläsionen ermöglicht.
- Sie kann dazu beitragen, charakteristische morphologische Merkmale im Falle von Genodermatosen zu erkennen.
- Die Dermatoskopie von Genodermatosen kann helfen, möglichst früh eine Diagnose zu stellen. Es ist sowohl für die Prävention als auch für die Therapie wichtig.
- Potenziell kann die Dermatoskopie zur Überwachung des therapeutischen Ansprechens eingesetzt werden.
